# Dictionary of Practical Medicine, Comprising General Pathology, the Nature and Treatment of Diseases, Morbid Structures, &c.

**Published:** 1846-08

**Authors:** 


ART. VII.—*Dictionary of Practical Medicine, comprising General Pathology, the Nature and Treatment of Diseases, Morbid Structures, &c.* By JAMES COPLAND, M. D., F. R. S. Edited with additions. By CHARLES A. LEE, M.D.

Part XVI of this admirable Dictionary has been for several weeks on our table. We congratulate the profession that the work is going forward with so much regularity, and that there is a prospect of its completion at no remote time. When brought to a close it will constitute a sort of encyclopædia of practical medicine with which no practitioner will willingly dispense. What has already been issued of it makes 1056 pages, with double columns in fine print.

